# Ecotoxicity assessment of dairy wastewater: environmental risks and regulatory needs

**DOI:** 10.1007/s10646-026-03088-7

**Published:** 2026-04-24

**Authors:** Magno Lange Albuquerque, Fernando Rodrigues-Silva, Alyson Rogério Ribeiro, Carollina M. Chávez, Gilcinéa de Cássia Santana, Alessandra da S. Martins, Camila C. Amorim, Maria Clara V. M. Starling

**Affiliations:** 1 Department of Sanitary and Environmental Engineering, Applied Ecotoxicology Group (ECOA), Escola de Engenharia, Universidade Federal de Minas Gerais, Av. Presidente Antônio Carlos 6627, Belo Horizonte, 31270-901 MG Brazil; 2 Department of Preventive Veterinary Medicine, Universidade Federal de Minas Gerais (UFMG), Av. Presidente Antônio Carlos 6627, Escola de Veterinária, Belo Horizonte, 31279-901 MG Brazil; 3 Department of Veterinary Clinic and Surgery, Universidade Federal de Minas Gerais, Av. Presidente Antônio Carlos 6627, Escola de Veterinária, Belo Horizonte, 31279-901 MG Brazil

**Keywords:** Acute toxicity, Chronic toxicity, *Daphnia similis*, *Raphidocelis subcapitata* and effluent

## Abstract

The dairy industry is the fourth largest in the food sector in Brazil, posing substantial environmental risks due to the discharge of dairy wastewater (DWW) into environmental waters. The numerous small-sized dairy facilities (500 − 30,000 L of milk processed day^− 1^) in the country are particularly concerning as they are subjected to less stringent environmental regulations. This study presents a comprehensive ecotoxicological analysis of DWW from a small dairy industry by exploring acute and chronic effects of the wastewater to test-organisms from various trophic levels and discussing results in light of current national and State-level regulations. Sampling was conducted during the dry and wet seasons. Acute and chronic toxicity were assessed for *Aliivibrio fischeri* (bacteria), *Raphidocelis subcapitata* (algae), *Daphnia similis* (crustacean), and *Danio rerio* (fish) larvae and adults. Acute toxicity tests revealed that DWW was toxic to all organisms in the following order: *Daphnia similis* (Toxicity Factor (TF) = 32) > *Danio rerio* (larvae) (TF ≥ 16) > *Danio rerio* (adult) (TF = 2). Bioassays with *Aliivibrio fischeri* showed great variability, probably due to matrix turbidity and color effects upon luminescence measurement, thus being the least appropriate species among those tested. Chronic toxicity tests showed the following outcomes: *Raphidocelis subcapitata* (TF = 32) > *Danio rerio* (larvae) (TF ≥ 16). Bioassays were also conducted with surface water from the DWW receiving pond and indicated a *hormesis* effect to bacteria and algae. *Raphidocelis subcapitata* and *Daphnia similis* were the most adequate organisms for chronic and acute ecotoxicity assessment, respectively. Results underscore the need to raise data on the toxicity of DWW, particularly those submitted to simplified treatment, and provides insights into the sensitivity of test organisms to this matrix, thereby contributing to the development of a more specific approach in industrial wastewater discharge regulations.

**Supplementary Information:**

The online version contains supplementary material available at 10.1007/s10646-026-03088-7.

## Introduction

Adverse environmental impacts caused by anthropogenic activities related to industrial production, including the dairy industry, are a subject of concern regarding pollution control measures. The primary environmental impact of the dairy industry is the intensive use of water, leading to a significant volume of wastewater (Akansha et al., 2020, Guerreiro et al. 2020). The dairy industry is the fourth-largest food sector in Brazil (Carvalho and Costa 2015), accounting for 27.6% of the total number of industries. Among Brazilian states, Minas Gerais (MG) is the leading producer of dairy products in the country. In 2022 and 2023, dairy industries in Minas Gerais processed 5,861,961 L and 5,786,613 L of raw milk, respectively (IBGE, 2024).

Dairy wastewater (DWW) discharge may cause public health issues and environmental quality impairment due to high nutrients and organic loads (Daneshvar et al. 2019). Besides, it is associated with dissolved oxygen depletion, strong odors, foam formation, fat layers, proliferation of disease vectors, and toxicity to aquatic species (Guerreiro et al. 2020). Even though, only a limited number of studies have analyzed the ecotoxicological effects of DWW to aquatic species, and data are even more scarce for small-sized industries, defined as those with production ranging from 500 − 30,000 L of milk processed day^− 1^ which are often eligible for simplified environmental licensing (Registration or Simplified Environmental Report - SER). This implies in less stringent procedural requirements than typically applied to large dairy facilities (Minas Gerais, 2017), such as simplified DWW treatment prior to discharge. Although self-monitoring and direct discharge of dairy wastewater (DWW) to receiving waters should only be allowed after due treatment, it may be waived for sources defined as low pollution potential, such as small-sized dairy facilities, by the competent environmental authority (Brasil, 2011).

Internationally, ecotoxicological screening has long been embedded in wastewater regulation to control ecological and human health risks of industrial wastewater discharge (Norberg-King et al., 2018). In the United States of America (USA), Whole Effluent Toxicity (WET) testing underpins discharge control by requiring standardized bioassays that capture the integrated effects of complex mixtures, rather than relying only on chemical-by-chemical limits (EPA, 2000). A similar rationale is supported in the European Whole Effluent Assessment (WEA), which combines lines of evidence to evaluate mixture toxicity in effluents and receiving waters (Convention for the Protection of the Marine Environment of the North-East Atlantic OSPAR Commission, 2007). In general, these frameworks operationalize compliance through dilution-based testing with multiple organisms and both acute and chronic endpoints, often reporting results as toxicity units (TU). Canada also advances in this logic by adopting sector-oriented programs, defining monitoring and testing schemes that better reflect the dominant stressors of specific industries, thereby improving sensitivity and interpretability (Goverment of Canada, 2012).

In Brazil, Directive No. 430/2011 establishes ecotoxicity testing with organisms from at least two trophic levels and states that effluents must not cause, nor have the potential to cause, toxic effects in receiving waters (Brasil, 2011), while Directive No. 357/2005 explicitly requires the absence of chronic toxicity for waters intended for species conservation (Brasil, 2005). However, current Brazilian directives remain comparatively generic regarding ecotoxicological assays and test-organisms to be used for wastewater from different industrial sources. Thus, it is critical to raise data to support the selection of the most appropriate assays for wastewater from different industrial sources aiming to avoid bias from tolerant organisms, as highlighted here for DWW (Alves et al. 2024).

Ecotoxicological assays evaluate acute or chronic effects of isolated substances and/or mixtures (i.e. effluents) on laboratory-cultured model organisms under controlled exposure conditions (Luan et al. 2020). Acute toxicity assays assess lethal effects reported as Effect Concentration (EC_50_) or Lethal Concentration (LC_50_) to 50% of the exposed population, or by Toxicity Factor (TF), defined as the dilution factor of the least diluted test concentration at which no effect was observed relative to the control (or No Observed Effect Dilution (NOED), typically reported as an integer Dilution Factor, DF) (Costa et al. 2008). Conversely, chronic toxicity refers to sublethal effects, such as morphological, reproductive, and behavioral changes, quantified by the Lowest Observed Effect Concentration (LOEC), Non-Observed Effect Concentration (NOEC), or TF.

In this context, considering: (i) the potential impacts of DWW discharge to aquatic biota, particularly for small-sized industries; (ii) the ecotoxicological approaches that enable the evaluation and quantification of acute or chronic effects of pollutants/effluents to aquatic biota; and (iii) the need to improve industrial wastewater discharge regulations in Brazil, this study performs a comprehensive characterization of acute and chronic ecotoxicity of DWW from a small-sized facility and surface water from the receiving pond. Observed toxic effects are clearly articulated with current regulations related to ecotoxicological criteria imposed to industrial wastewater discharge in Brazil, particularly for dairy wastewater. Due to limited access to wastewater from various facilities, the investigation was conducted as an in-depth case study of a small-sized dairy plant subjected to simplified licensing and discharge procedures, among the many operating under similar regulatory circumstances in Minas Gerais. Seasonal field sampling combined with a multi-trophic battery of acute and chronic bioassays provides a structured baseline to interpret DWW toxicity, identify sensitivity hierarchy patterns among test organisms, and evaluate matrix-related constraints affecting assay performance. By maintaining internal methodological consistency across sampling campaigns, the study allows for robust comparison of organism response and seasonal variability within the same operational context. Finally, by integrating ecotoxicological endpoints with the Brazilian regulatory framework for industrial wastewater discharge, this study translates field-based toxicity data into discharge-compliance scenarios, thereby contributing with technically sound data to improve the dialogue between academia and environmental authorities and support the refinement of ecotoxicity-based discharge regulations for DWW.

## Materials and methods

### Case study: diary industry

DWW and surface water from the receiving pond were sampled in a small-sized dairy industry located in Esmeraldas/MG, Brazil. The dairy industry has been operating for ten years and processes 1,200 L of milk per day to produce fresh cheese and yogurt (seasonal production). Its main production stages for cheese and yogurt manufacturing and principal wastewater generation points are presented in the Supplementary Material (SM) (Figure S1).

The average generation of DWW by dairy industries with similar production processes ranges from 0.2 to 10 L kg^− 1^ of processed milk (Akansha et al., 2020; Giner Santoja et al. 2019; Guerreiro et al. 2020). For this industry, DWW generation was estimated at approximately 3,083.16 L day^− 1^ (calculation details in the SM, Eq. S1). As this is a small industry classified as a low-pollution potential facility, it is subjected to less stringent pollution control requirements. Hence, the wastewater treatment consists of a standalone oil and grease chamber, followed by wastewater discharge in a pond (2,325 m^2^, surface water Class 2 (Brasil, 2005). This is the main treatment applied in similar small-sized dairy facilities in the state of Minas Gerais.

Two sampling campaigns were conducted, one on August 9, 2023 (during the dry season, April – September) and the other on January 31, 2024 (during the rainy season, October – March). A grab sampling scheme was applied for DWW (output of the oil and grease chamber) to achieve a representative sample, with hourly subsamples collected from 9:00 am to 12:00 pm during cheese manufacturing cycles. Surface water sampling from the pond that receives the DWW was carried out as a discrete sample at the DWW discharge point. Both samples were collected on the same day following the Standard Methods for the Examination of Water and Wastewater (APHA, 2023).

### Physicochemical characterization

Samples were stored at 4 °C until characterization, which was carried out within 24 h of sampling. Physicochemical characterization was performed following the Standard Methods for the Examination of Water and Wastewater (APHA, 2023) for alkalinity (2320-B), biochemical oxygen demand (BOD, 5210-B), true color (2120-C), chemical oxygen demand (COD, 5220-D), dissolved oxygen (4500-O-G), hardness (2340-C), pH (4500-H^+^-B), orthophosphate (4500-P-E), total nitrogen (4500-N-C), total solids (TS, 2540-B), total dissolved solids (TDS, 2540-C), total suspended solids (TSS, 2540-D), turbidity (2130-B). Following characterization, samples were frozen before ecotoxicological assays according to NBR 15,469 (ABNT, 2021).

### Ecotoxicological assays

Ecotoxicological assays were performed based on guidelines from the Brazilian Association of Technical Guidelines (ABNT) presented in Table 1.

**Table 1:** Standard assays used for ecotoxicological characterization of dairy wastewater and surface water collected at the wastewater discharge point in the receiving water body

Standard	Test organism	Trophic level	Exposure period	Endpoint
ABNT. NBR 12,648/2023: Aquatic ecotoxicology – Chronic toxicity – Test with algae (Chlorophyceae)	*Raphidocelis subcapitata*	Producer	From 72 to 96 ± 2 h	Reproduction and morphology
ABNT. NBR 12,713/2022: Aquatic ecotoxicology – Acute toxicity – Test with *Daphnia spp* (Cladocera, Crustacea)	*Daphnia similis*	Primary consumer	24 and 48 h	Mobility inhibition and lethality
ABNT. NBR 15,088/2022: Aquatic ecotoxicology – Acute toxicity – Test with fish (Cyprinidae)	*Danio rerio*	Secondary consumer	48 h	Mobility inhibition and lethality
ABNT. NBR 15,411/2021: Aquatic Ecotoxicology – Inhibitory effect on V*ibrio fischeri* bioluminescence Part 3: method using lyophilized bacteria	*Aliivibrio fischeri*	Decomposer	5, 15 and 30 min	Bioluminescence decay
ABNT. NBR 15,499/2022: Aquatic Ecotoxicology: Short term chronic toxicity – Test with fish	*Danio rerio*	Secondary consumer	72 and 168 h	Growth and mobility inhibition, and lethality

#### *Raphidocelis subcapitata* – chronic toxicity

The green algae *Raphidocelis subcapitata* was granted by SENAI Center of Innovation and Technology (Minas Gerais, Brazil) and bred in the lab. The chronic effect analyzed in this assay is the inhibition of algae biomass growth compared to the control. For the ecotoxicity analysis, bioassays were carried out in triplicates of blanks (0% of the sample) and eight different dilutions of DWW with culture medium Oligo (100%, 50%, 25%, 12.5%, 6.25%, 3.125%, 1.562%, 0.781%, v: v) in glass *Erlenmeyer* flasks (150 mL). The assay was inoculated with resuspended cells, and all the test recipients were placed in a horizontal shaker under standard and constant rotation conditions (150 rpm), temperature (25 °C), and luminosity (4500 lx–24 h), for 72 ± 2 h. After removing samples from the shaker, they were isolated in encoded containers and preserved with a Lugol solution (4 g of Metallic Iodine: 6 g of Potassium Iodide) within a week. An aliquot (50 µL) of sample from each dilution was taken and placed in a *Neubauer* chamber under an optical microscope (Nikon i50) for cell counting to allow for biomass calculation as detailed in the Supplementary Material (Eq. S2). Data was analyzed using ToxStat software and results were reported as NOEC and TF. Sensitivity Assays were carried out with Sodium Chloride (NaCl) solutions, and the Control chart is available in the Supplementary Material File (Figure S2).

#### *Daphnia similis* – acute toxicity

The microcrustacean *Daphnia similis* was granted by the SENAI Center of Innovation and Technology (Minas Gerais, Brazil) and bred in the lab. Organisms were cultured in glass containers with MS medium Under controlled temperature (20 ± 2 °C) and photoperiod (16 h light/8 h dark) keeping a density of 0.025 org/mL. Organisms were fed daily with *Raphidocelis subcapitata* and weekly with yeast and solubilized fish feed. For the bioassay, neonates (maximum 24 h old) were collected from a stock of daphnid clones. Ecotoxicity analysis was carried out in quadruplicates (10 mL) of blanks (0% of the sample) and six dilutions of DWW with the culture medium (100%, 50%, 25%, 12.5%, 6.2%, 3.1%, v: v). Afterward, pH and dissolved oxygen were measured for each replicate. Five neonates (≤ 24 h) were added to sterile centrifuge tubes of plastic containing each sample, and these flasks were incubated in the dark at 20 ± 2 °C. Replicates were visually monitored after 24 and 48 h of exposure to count the number of immobile organisms. Sensitivity assays were conducted using sodium chloride (NaCl) solutions, and the control chart is presented in the Supplementary Material (Figure S3). Data were analyzed using OriginPRO software through the Dose Response and Trimmed Spearman-Karber methods, and results were reported as 48 h LC_50_ and TF for sensitivity and DWW, respectively.

#### *Danio rerio* (adult) – acute toxicity

The adult *Danio rerio* (Zebrafish) were granted by CETESB and bred in the lab. *Danio rerio* (2.0 ± 1.0 cm) were cultivated in polycarbonate aquariums filled with reconstituted water at a ratio of 1 g of organism L^− 1^ under standard conditions of pH (6.5–7.5), temperature (23 °C), dissolved oxygen (5 mg L^− 1^), and hardness (10–60 mg CaCO_3_ L^− 1^) and a photoperiod of 12 h/12 h. Sensitivity Assays were carried out with Sodium Chloride (NaCl) solutions and the control chart is available in the Supplementary Material File (Figure S4). For the assay setup, healthy fish were selected, and ten of these organisms were randomly added to each of the quadruplicates of blanks (0%) and five sample dilutions of DWW with reconstituted water (100%, 50%, 25%, 12.5%, 6.25%, v: v), and a positive control with NaCl (lethal concentration). The bioassay was carried out for 48 h following the standard conditions applied for cultivation. Organisms were fed twice a day with commercial feedstuff (45% protein) and recently hatched *Artemia sp.* Mortality, behavioral, and morphological changes were assessed daily. Data were analyzed using R software, and results were reported as NOEC and TF.

#### *Danio rerio* (larvae) – acute and chronic toxicity

Sensitivity Assays were carried out with Sodium Chloride (NaCl) solutions as shown in the control chart available in the Supplementary Material File (Figure S5). A day before the assay was carried out, *Danio rerio* male and female were segregated in a continuous renovation flow system in a ratio of 2 males per female (2:1) during the night. Deposited eggs were collected the following morning, cleaned with reconstituted water, and set in a petri dish to be selected for ecotoxicological assays with a stereoscopic magnifier (≥ 80 x). Fertilized larvae were set in an incubator (Scienlabor, Zebrafish LARVAE UV) with a 14 h light/10 h dark photoperiod. After hatching, larvae were segregated again and those which presented abnormalities were discarded. Bioassays were carried out in quadruplicate (250 mL) of blanks (0%), and five sample dilutions of DWW with reconstituted water (100%, 50%, 25%, 12.5%, 6.25%, v: v), and positive control with NaCl (lethal concentration as ecotoxicological control chart). Conductivity, pH, and dissolved oxygen were measured before the bioassay and ten larvae (< 24 h) were randomly distributed in each replicate. The assay was carried out for 168 ± 1 h in an incubator at 25 ± 2 °C and a photoperiod of 14 h light/10 h dark. Mortality and morphological abnormalities were assessed daily and expressed as percentages for each sample. Test solutions were renewed twice, and for each renewal, conductivity, pH, and dissolved oxygen were measured for previous and new test solutions prior to larvae recipient rearrangement. Data were analyzed using R software, and results were reported as NOEC and TF.

#### *Aliivibrio fischeri* – acute toxicity

Lyophilized bacteria *Aliivibrio fischeri* were acquired from Modern Water. The test setup began with sample salinity adjustment with NaCl to ≥ 20% (m: v). The bioassay was carried out using the Microtox^®^ LX analyzer (Modern Water). The sample was prepared by a dilution series in duplicates of blanks (0%) and nine different dilutions of DWW with NaCl solution at 2% (m: v) in glass cuvettes (81.9%, 40.95%, 20.475%, 10.237%, 5.118%, 2.559%, 1.279%, 0.639%, 0.319%, v: v). Finally, samples were analyzed for bacteria bioluminescence inhibition at 5-, 15-, and 30-min. Data were analyzed using the Microtox Analyzer Software, and results were reported as EC_50_. Sensitivity assays of the stock culture organisms were conducted using potassium dichromate (K_2_Cr_2_O_7_, purity ≥ 93,5%) as a reference substance. The control chart is available in the Supplementary Material File (Figure S6).

### Critical analysis of current toxicity standards

Results obtained from ecotoxicological assays performed in this study were expressed as NOEC, TF, LC_50_, and EC_50_ and discussed in light of current national (Brazil) and state level environmental requirements related to ecotoxicity of industrial wastewater and surface water. Briefly, directive CONAMA No. 430/2011 (Brasil, 2011) establishes two methodologies for assessing the maximum concentration of wastewater allowed in freshwater bodies. These methodologies rely on the reported NOEC values for chronic toxicity, and TF and/or LC_50_ for acute toxicity. For water bodies classified as Classes 1 and 2 (Brasil, 2005), the maximum wastewater concentration (Equation S3) allowed must be less than or equal to the NOEC derived from a chronic toxicity test. For acute toxicity assessment, Equations S4 and S5 determine the maximum wastewater concentration allowed. However, for Class 3 freshwater bodies (Brasil, 2005), Equations S6 and S7 are used to assess the maximum allowable wastewater concentration. Additionally, Directive FATMA No. 17/2002 (Santa Catarina, 2002) determines the maximum wastewater concentration in freshwater bodies based on TF values from acute toxicity responses (Equation S8).

## Results and discussion

### Physicochemical characterization of dairy wastewater and surface water samples

Table 2 presents the physicochemical characterization of DWW and surface water sampled at the small-sized dairy industry located in Esmeraldas/MG compared to typical values reported in the literature (Patil and Kurhekar 2021; Tabelini et al. 2023; and CETESB and FIESP 2006), and maximum values imposed for wastewater discharge into water bodies (Minas Gerais, 2022).

The DWW is white in color and shows visual signs of oil and grease. COD values for DWW sampled in the rainy season were 32,31 to 92,60% lower than the values typically reported in the literature for this wastewater. Despite lower values, COD concentrations in the dry season sample were within the usual range reported by CETESB and FIESP (2006). However, COD values did not comply with the maximum concentration (≤ 180 mg L^− 1^) established by the state regulation (Minas Gerais, 2022). COD and BOD_5_ levels indicate a high presence of organic pollutants which may result in oxygen depletion when discharged into surface water, as observed for the rainy season. This outcome can contribute to increased toxicity levels (Diaz-Sosa et al. 2020). Besides, the BOD_5_/COD ratio (< 1) calculated for the DWW in both seasons indicates that the effluent is biodegradable (Herrera et al. 2024). Considering the estimated flow of DWW discharge (3,083 L per day^− 1^), the input load of total nitrogen and phosphorus in the receiving water body corresponds to 174.51 g per day^− 1^ and 132.88 g per day^− 1^, respectively. These loads may contribute to the eutrophication of the receptor water body (Wang et al. 2020), a condition that may affect ecosystem functioning and toxicity to aquatic biota.

**Table 2:** Physicochemical characterization of dairy wastewater and receiving surface water sampled during dry and rainy seasons from a small-sized dairy industry in Minas Gerais, Brazil, typical values reported in the literature, and national and state regulation standards

Parameters	Dairy wastewater						Surface water		
	DWW (dry season)	DWW (rainy season)	Patil and Kurhekar (2021)	Tabelini et al. (2023)	CETESB and FIESP (2006)	COPAM/CERH No. 08/2022 (Class 2 wastewater discharge)	Surface water (dry season)	Surface water (rainy season)	CONAMA No. 357/2005 (Class 2)
Alkalinity (mg CaCO_3_ L^-1^)	6.00 ± 3.20	0.0	-	-	-	-	4.13 ± 1.38	382.50 ± 5.30	-
BOD_5_ (mg O_2_ L^-1^)	689.17 ± 5.43	293.47 ± 8.55	641	1914	450–4790	≤ 60	63.37 ± 1.58	33.33 ± 2.14	≤ 5
COD_soluble_ (mg O_2_ L^-1^)	791.00 ± 87.70	338.42 ± 0.74	2026	4575	500–4500	≤ 180	1,798.75 ± 65.41	929.90 ± 0.43	-
BOD_5_/COD	0.87	0.86	-	-	-	-	-	-	-
Color (mg PtCo L^-1^)	114.50 ± 0.70	29.50 ± 2.12	-	-	-	-	100.00 ± 2.83	233.50 ± 28.99	≤ 75
Hardness (mg L^-1^)	90.43 ± 21.73	98.24 ± 6.41	-	-	-	-	59.45 ± 8.55	72.04 ± 2.14	-
Dissolved oxygen (mg L^-1^)	-	-	-	-	-	-	4.56	0.48	≥ 5
pH	4.4	4.3	7.7	-	5.3–9.4	5–9	6.8	6.3	6–9
Orthophosphate (mg L^-1^)	53.10	56.60	-	15.6		-	44.50	40.20	-
Total Nitrogen (mg L^-1^)	52.15	43.10	-	139	15–180	-	31.40	23.80	~ 13
TSS (mg L^-1^)	1790.00 ± 33.00	1862.50 ± 66.17	293	24–5700		≤ 1050	193.33 ± 10.14	≤ 100	-
TDS (mg L^-1^)	520 ± 11.42	317.50 ± 38.89	1078	-		-	660.30 ± 18.99	-	≤ 500
TS (mg L^-1^)	2,351.00 ± 22.63	2,198.50 ± 7.99	1421	135–8500		≤ 3938	1,049.50 ± 13.92	-	-
Turbidity (NTU)	298.00 ± 5.66	293.00 ± 4.24	808		258.50 ± 2.12	≤ 1530	-	-	≤ 100

BOD5: Biochemical Oxygen Demand measured over five days; COD: Chemical Oxygen Demand; CODsoluble: Soluble Chemical Oxygen Demand; TSS: Total Suspended Solids; TDS: Total Dissolved Solids; TS: Total Solids; NTU: Nephelometric Turbidity Unit; PtCo: Platinum-Cobalt color unit; CETESB: Environmental Company of the State of São Paulo; FIESP: Federation of Industries of the State of São Paulo; COPAM: Environmental Policy Council of Minas Gerais; CERH: State Water Resources Council; CONAMA: National Environment Council of Brazil; DWW: Dairy Wastewater

BOD_5_: Biochemical Oxygen Demand measured over five days; COD: Chemical Oxygen Demand; CODsoluble: Soluble Chemical Oxygen Demand; TSS: Total Suspended Solids; TDS: Total Dissolved Solids; TS: Total Solids; NTU: Nephelometric Turbidity Unit; PtCo: Platinum-Cobalt color unit; CETESB: Environmental Company of the State of São Paulo; FIESP: Federation of Industries of the State of São Paulo; COPAM: Environmental Policy Council of Minas Gerais; CERH: State Water Resources Council; CONAMA: National Environment Council of Brazil; DWW: Dairy Wastewater.

TSS values exceeded environmental standards in both sampling campaigns, indicating the need for solids removal from DWW prior to discharge. This may be a consequence of the simplified wastewater treatment (oil and grease chamber only) applied in the facility, as pollution control requirements are less stringent for small-sized dairy facilities which present low pollution potential. However, suspended solids may lead to effects on filtrating organisms (Weltens 2000) and fish by promoting the clogging of gills (Montoya et al. 2024). In addition, DWW was more acidic (pH 4.4 and 4.3) compared to other studies and does not comply with wastewater discharge standard set by Minas Gerais (2022), suggesting the need for neutralization prior to discharge as water pH is a critical factor for protein activity as well as contaminants bioavailability, thus influencing aquatic life (Zhao et al. 2024; Di Russo et al. 2012).

Despite the discharge of DWW, the receiving water pH remained near neutral (Table 2), indicating its capacity to buffer wastewater’s acidity. The decline in dissolved oxygen levels in the surface water of the receiving pond during the rainy season is potentially associated with the discharge of organic matter present in DWW. Additional loads may also contribute to surface water alkalinity increase during the rainy season, due to the intake of Mg^2+^ and Ca^2+^, as reflected in alkalinity and hardness values shown in Table 2 (Middelburg et al. 2020). Other parameters followed the usual values observed in the literature for DWW.

Considering the physicochemical characterization of DWW and surface water shown in Table 2, the main concerns for ecotoxicity are related to total nitrogen and phosphorus, for which high concentrations were reported elsewhere (Abbas et al. 2018). These nutrients may lead to surface water eutrophication, a state that increases toxicity due to the possibility of toxic algae blooms (Paerl et al. 2001). In addition, the cascade of events following nutrient increase may contribute to oxygen depletion and pH decrease, especially in the deep waters and at night. These conditions impact groups that are sensitive to low oxygen concentrations, such as fish, and increase the solubility and bioavailability of toxic compounds (i.e. metals), as well as the production of toxic sulfur compounds (i.e. H_2_S) produced via anaerobic pathways (Guan et al. 2023).

### Ecotoxicity of dairy wastewater and surface water samples

Table 4 presents acute and chronic ecotoxicity responses obtained from the exposure of test organisms from different trophic levels to DWW and receiving surface water sampled during dry and rainy seasons from a small-sized industry in the state of Minas Gerais.

#### Dairy wastewater ecotoxicity

All acute toxicity assays performed with DWW presented effects at some dilution factors, except for *Aliivibrio fischeri* in the dry season sample, probably due to incompatibility between sample physicochemical characteristics and testing endpoint. Among the four test organisms used for acute toxicity bioassays, higher toxicity was observed for *Daphnia similis* in both campaigns as effects to this organism were observed up to concentrations of 3.125% DWW. Effects on *Danio rerio* larvae were still observed at 6.25% DWW, the most diluted sample evaluated in these bioassays (ABNT, 2022). On the other hand, no effects upon adult *Danio rerio* were observed at concentrations below 50%.

Regarding *Aliivibrio fischeri*, DWW sample obtained in the dry season was not toxic, yet the wet season sample was highly toxic (Table 3). This variability did not occur for any of the other tests and may be related to the physiochemical profile of the matrix, as the sample from the dry season showed high color and turbidity and these features may influence luminescence measurements compromising the bioassay (Ma et al. 2014). In addition, the sample used for this toxicity assay is prepared in 2% NaCl to mimic a seawater environment for the marine bacteria used as test-organism. Gao et al. (2012) discuss that saline adjustment conditions during preparation may change the characteristics of the tested sample by decreasing metals bioavailability and the solubility of organic substances. Hence, even though *Aliivibrio fischeri* is highly recognized by environmental agencies worldwide and has been used to determine the acute toxicity of pesticides (Tóth et al. 2019), metals (Tsiridis et al. 2006), pharmaceutical drugs (Santos et al. 2024), industrial wastewater (Butarewicz et al. 2019), and municipal wastewaters before and after advanced treatments (Rodrigues-Silva et al. 2024), it was considered unsuitable for the ecotoxicological evaluation of this DWW.

**Table 3:** Ecotoxicological characterization of dairy wastewater and receiving surface water sampled during dry and rainy seasons campaigns from a small-sized dairy industry in Minas Gerais, Brazil according to the toxicity factor

Sample	Acute toxicity (TF)				Chronic toxicity (TF)	
	Aliivibrio fischeri^(a)^	Daphnia similis	Danio rerio (adult)	Danio rerio (Larvae)	Raphidocelis subcapitata	Danio rerio (Larvae)
DWW dry season	NT	32	2	≥ 16	32	≥ 16
DWW rainy season	0.86	32	2	≥ 16	32	≥ 16
Surface water dry season	NT	^(b)^	^(b)^	^(b)^	16	^(b)^
Surface water rainy season	NT	NT	NT	8	128	8

DWW: Dairy Wastewater; TF: Toxicity Factor; NT: Non-toxic. ^(a)^Results reported as EC_50_(%) – 30 min. ^(b)^Bioassays were not carried out

Despite the fast and practical operation of the *Aliivibrio fischeri* assay, its use as a standard test organism for wastewater discharge requires further investigation on a case-by-case basis, especially considering colorful effluents. A study by Ramírez-Morales et al. (2024) also reported variable sensitivity between test organisms in the assessment of DWW toxicity. While their study reported that *Aliivibrio fischeri* was more sensitive to DWW, our findings highlight its unsuitability for ecotoxicological evaluation of DWW samples. This discrepancy suggests that sample physicochemical characteristics can significantly influence toxicity outcomes, requiring further research on the toxicity of DWW produced across different conditions, and emphasizing the need case-by-case evaluation. Non-monotonic responses have also been reported for bioluminescent bacteria exposed to industrial wastewater, where low-level exposure to complex environmental samples can stimulate luminescence before inhibition occurs (Agathokleous et al. 2024). These *hormesis* patterns are thought to arise from interactions between test chemicals and luciferase biochemistry as well as from the influence of nutrient and ion composition on enzyme activity.

Regarding chronic toxicity, *Raphidocelis subcapitata* was the most sensitive test organism, showing the highest toxicity values for DWW matrix in both sampling campaigns. Concentrations greater than 3.125% of DWW can cause growth inhibition and morphological alterations in this species. Similarly, Ramírez-Morales et al. (2024) also demonstrated the effect of DWW on producers, therein represented by *Lactuca sativa*, for which there was high inhibition of seed germination (> 90%).

DWW sampled in both seasons promoted measurable effects on *Danio rerio* larvae at concentrations ≥ 6.25%, whereas adult fish exhibited lower sensitivity. This ontogenetic difference aligns with ecotoxicological evidence that early life stages are more vulnerable to complex contaminant mixtures due to incomplete physiological development, higher metabolic rates, and reduced detoxification capacity (Kovrižnych et al. 2013). Larval sensitivity has implications beyond individual-level endpoints, as impairment during early developmental stages may compromise recruitment success and long-term population stability.

Across trophic levels, the highest sensitivities were observed for primary producers (*Raphidocelis subcapitata*) and primary consumers (*Daphnia similis*), a pattern that is consistent with studies showing that nutrient-rich industrial effluents often exert disproportionate stress at the base of the food web. Because algae and cladocerans form the foundation of aquatic trophic networks, their sensitivity suggests that DWW discharge, even after partial dilution, may alter primary productivity, grazing dynamics, and energy transfer efficiency, potentially propagating indirect effects to higher trophic levels. As *Daphnia similis* (acute toxicity) and *Raphidocelis subcapitata* (chronic toxicity) were the most responsive organisms for DWW evaluation regarding both acute and chronic endpoints, the inclusion of lower-trophic-level organisms as early-warning indicators in WET-based monitoring frameworks is recommended. This may avoid the underestimation of ecotoxicological risks by relying solely on bacterial luminescence assays or adult-fish mortality.

From a regulatory perspective, DWW ecotoxicity results obtained in this study demonstrate that organism selection and dilution design are not merely methodological decisions but determining factors in compliance classification under toxicity-based standards. Depending on which organism drives the lowest no-effect dilution, the same effluent may be categorized differently under state-level TF thresholds. Therefore, ecologically informed organism selection is essential for regulatory assessment and should be explored in the improvement of wastewater discharge policies to support environmental risk control.

Moreover, to ensure conclusive ecotoxicity assessments, it is essential to include organisms from multiple trophic levels, as responses can vary widely depending on the ecological and seasonal context. Overall, the sensitivity pattern observed herein supports a precautionary interpretation of WET data for DWW in which endpoints driven by primary producers and cladocerans may provide earlier signals of ecological impairment than bacterial bioluminescence or adult-fish survival alone. Therefore, organism selection and dilution design should not be seen as mere methodological choices, but as regulatory determinants as they directly influence decision making on discharge scenarios compliance under toxicity-based standards.

#### Surface water from the recipient pond ecotoxicity

Two test organisms were used to assess the toxicity of surface water sampled from the pond where the DWW is discharged in the rainy season (Table 3), while four organisms were used for samples obtained in the wet season. No acute toxicity of surface water was observed for *Aliivibrio fischeri* in any of the seasons, and no acute effects were detected for *Daphnia similis* nor adult *Danio rerio* for samples obtained in the rainy season. In contrast, acute toxicity to *Danio rerio* larvae (TF = 8; no effect observed at 12.5% of the surface water sample) was observed for this sample.

Chronic toxicity of surface water samples was assessed using *Raphidocelis subcapitata* in both seasons and *Danio rerio* larvae for the rainy season sample. The microalgae bioassay revealed non-monotomic (*hormesis*-like) responses in both sampling campaigns. According to Agathokleous et al. (2025), *hormesis* is defined as a biphasic dose-response characterized by low dose stimulation and high-dose inhibition. In the context of wastewater-impacted receiving waters, *hormesis*-like responses may arise from compensatory growth stimulation at moderate dilutions where nutrients remain bioavailable but inhibitory contaminants are partially diluted. Chapman (2002) associates the *hormesis* effect in toxicity assays with the presence of high nutrient levels in surface water samples. This is the case of the pond receiving DWW in this case-study, as it is a eutrophic ecosystem (nitrogen: 23.80–31.40 mg L^− 1^; phosphorous: 40.20–44.50 mg L^− 1^). Under such conditions, nutrient assimilation may stimulate algal growth at intermediate dilutions, whereas higher concentrations introduce inhibitory effects associated with organic load, ammonia, or other components present in the mixture. This interaction between nutrient-driven stimulation and contaminant-induced inhibition complicates conventional monotonic dose–response modeling. From a regulatory standpoint, these findings suggest that single dilution testing or reliance on a single endpoint may fail to capture ecologically meaningful responses in eutrophic systems. Thus, multi-dilution designs and multi-endpoint approaches should be required to characterize ecotoxicological risks in such complex systems. Besides, it should be recognized that the *hormesis* effect detected in this bioassay corresponds to the overgrowth of producers, which affects the ecological balance in the receiving waterbody.

**Fig. 1:**
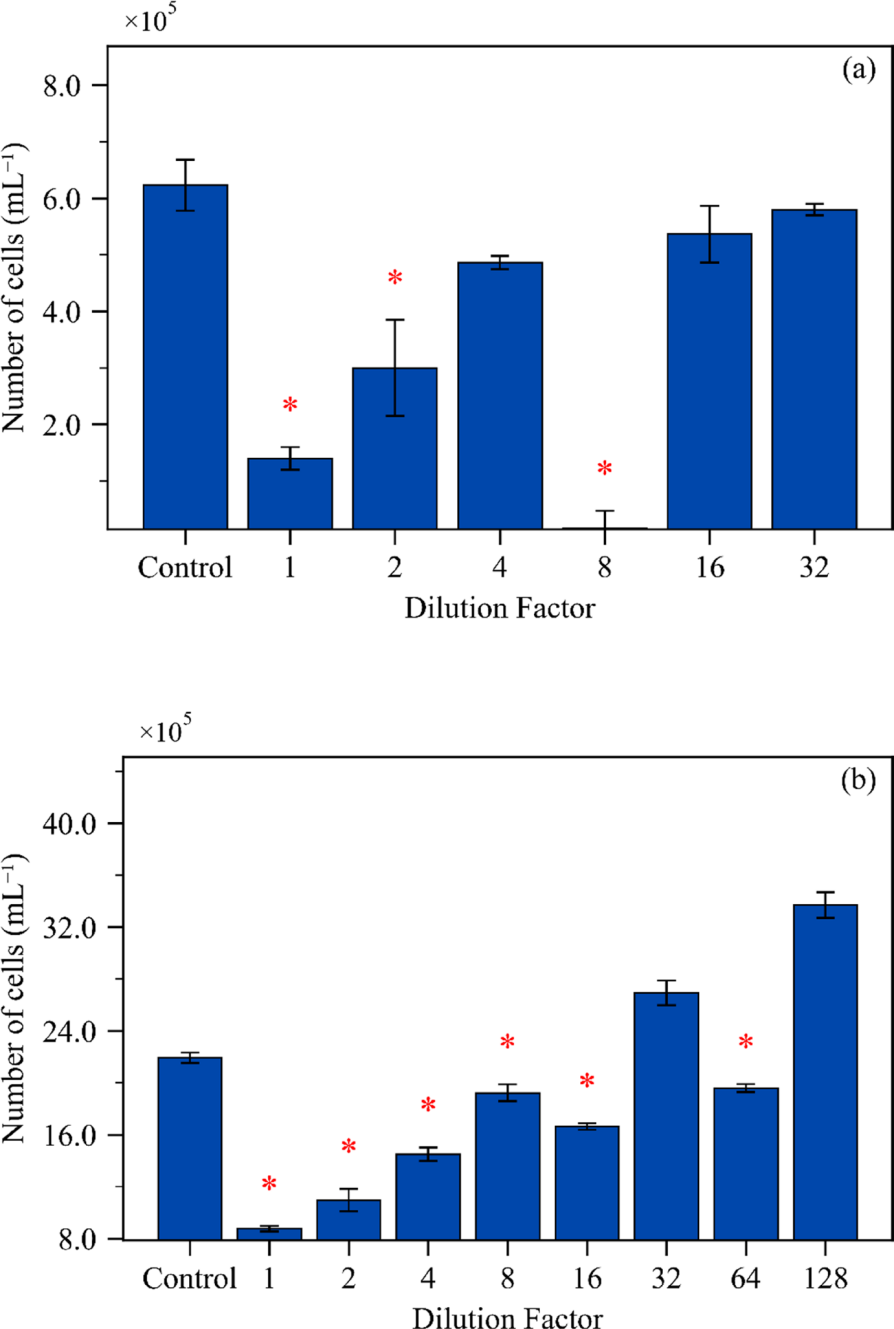
Average *Raphidocelis subcapitata* cell density after exposure to surface water from the receiving pond sampled in the (**A**) dry and (**B**) rainy seasons, compared to the control (mean ± standard deviation, *n* = 3). Statistical analysis was performed using Dunnett’s test (α = 0.05) in the software ToxStat. Coefficient of variation ≤ 0.1

Statistical analysis of algae biomass growth during exposure to the DWW sample showed that lower dilution factors in the dry season resulted in chronic toxicity (TF = 2; 50% of the sample). In contrast, dilution factors of 4 (25% of the sample), 16 (6.25% of the sample), and 32 (3.12% of the sample) showed no significant difference (⍺ = 0.05) when compared to the test control, and a dilution factor of 8 (12.5% of the sample) resulted in chronic toxicity (Fig. 1A). This was also verified for samples obtained in the rainy season, for which a dilution factor of 16 was toxic (TF = 16; 6.25% of the sample), yet dilution factors of 32 (3.12% of the sample) and 128 (0.78% of the sample) showed no toxicity, and the dilution factor of 64 (1.56% of the sample) resulted in chronic toxicity (Fig. 1B). Such patterns are characteristic of mixture-exposure systems where nutrient availability, contaminant speciation, and physicochemical conditions (e.g., pH, conductivity) interact to influence bioavailability and ecotoxic dynamics. Within the eutrophic receiving pond, continuous nutrient enrichment from DWW discharge likely modulates toxicity expression across seasons, partially explaining the variation in TF values between campaigns. These dynamics reinforce the relevance of seasonal testing and caution against extrapolating toxicity from single-event sampling. Overall, the integration of trophic-level sensitivity, ontogenetic vulnerability, seasonal variability, and regulatory threshold interpretation presented herein demonstrates how field-derived WET data can support the refinement of industrial wastewater discharge guidelines.

The difference in TF values between sampling campaigns is notably associated with a *hormesis* effect to algae, demonstrating how it affects traditional toxicity assessments, challenges standard dose-response models, and reinforces the need for alternative analytical methods and the use of multiple ecotoxicological assays. Despite complications promoted by nutrients present in surface water in the bioassay with microalgae, this organism is still more sensitive than *Danio rerio* (adult and larvae).

### Current wastewater discharge regulation and dairy wastewater toxicity: insights from the case-study

Ecotoxicity requirements for industrial wastewater discharge are heterogeneously implemented across Brazil, with only 6 of 27 federal units establishing previsions that extend beyond the general national framework (Table 4). While most state regulations formally adopt Directive CONAMA No. 430/2011 requirements that establish the use of acute and/or chronic bioassays for at least two organisms from different trophic levels (Brasil, 2011), they rarely provide typology-oriented guidance for selecting test organisms according to wastewater characteristics. This regulatory gap limits inter-study comparability hampers the development of harmonized toxicity databases and weakens the scientific basis for refining discharge criteria. In contrast, some Brazilian states incorporate operational elements, such as discharge-volume-based sampling frequency (e.g., Rio Grande do Sul) or predefined test-organism lists and TF thresholds (e.g., Rio de Janeiro and Santa Catarina), including sector-specific references to DWW. However, such instruments vary considerably in scope, terminology, and stringency. Table 4 summarizes the applicable federal and state-level frameworks, designated test organisms, and relevant toxicity thresholds to contextualize the ecotoxicological outcomes observed in this study.

Among the state-level instruments, Paraná and São Paulo provide the most structured guidance for organism selection. Paraná establishes sector-oriented acute and chronic effect requirements and sets TF = 2 as a regulatory threshold for DWW, whereas São Paulo adopts case-specific evaluation under CETESB technical standards, recommending *Daphnia similis* (acute) and *Ceriodaphnia dubia* (chronic) as test-organisms along with complementary assays (*Aliivibrio fischeri* and *Raphidocelis subcapitata*) depending on wastewater characteristics. In parallel, surface water governance in Brazil (Brasil, 2005) establishes class-dependent toxicity restrictions (chronic toxicity prohibited in Classes 1 and 2; acute toxicity prohibited in Class 3), and Minas Gerais further requires the absence of both acute and chronic toxicity in freshwater Classes 1–3. Collectively, these provisions indicate that DWW discharge should not induce measurable toxicity in receiving waters. Compliance with this requirement would vary for the DWW analyzed in this study according to (i) the bioassay used to assess matrix toxicity, (ii) tested dilution factors, and (iii) seasonality sampling scheme. This reinforces the importance of selecting appropriate test-organisms and interpreting organism sensitivity patterns and TF thresholds in light of matrix-specific limitations identified in this study.

**Table 4:** National and state regulations that enforce ecotoxicity standards for industrial wastewater discharge into surface water in Brazil

Country/State	Regulation	Test organisms	Main points
Brazil	Directive CONAMA No. 357, 17/03/2005	Not specified	Requires the absence of acute (Class 1, 2, 3) and chronic (Class 1, 2) toxicity in surface water
	Directive CONAMA No. 430, 05/13/2011	Organisms from at least two trophic levels (not specified)	The effluent should not promote acute or chronic toxicity to the receiving water body as according to its classification
Minas Gerais	Joint Normative Deliberation COPAM/CERH No. 08, 11/21/2022	Organisms from at least two trophic levels (not specified)	Freshwater bodies classified as classes 1, 2 or 3 must not exhibit acute nor chronic ecotoxicity
Rio Grande do Sul	Decree FEPAM No. 66, 12/19/2017	Organisms from at least two trophic levels (not specified)	Introduces criteria for sampling frequency based on the volume of wastewater discharge
Rio de Janeiro	Directive CONEMA No. 86, 12/07/2018	*Danio rerio*, *Pimephales promelas*, *Daphnia spp.*, *Aliivibrio fischeri*	Outlines test organisms for acute toxicity; set a threshold for Toxicity Factor (TF > 4)
Santa Catarina	Decree FATMA No. 017, 04/18/2002	*Daphnia magna*, *Aliivibrio fischeri*	Defines maximum acute toxicity for each industrial sector; sets threshold values for DWW toxicity based on *Daphnia magna* (TF = 2) and *Aliivibrio fischeri* (TF = 4).
Paraná	Directive CEMA No. 81, 10/19/2010	*Daphnia magna*, *Aliivibrio fischeri*, *Ceriodaphnia dubia*, *Scenedesmus subspicatus*	Defines acute and chronic test organisms according to the industrial sector; sets threshold values for DWW (TF = 2) for all test organisms
São Paulo	Directive SMA No. 003, 02/22/2000	*Daphnia similis*, *Ceriodaphnia dubia*, *Aliivibrio fischeri*, *Raphidocelis subcapitata*	Selects test organisms on a case-by-case basis, recommends acute and chronic bioassays based on wastewater characteristics

Note: Surface water classes: Special (water supply, aquatic balance, and conservation areas); Class 1 (water supply after simplified treatment, aquatic protection, primary recreation, and irrigation), Class 2 (water supply after conventional treatment, aquaculture, and broader uses), Class 3 (water supply after advanced treatment, and secondary uses), and Class 4 (for navigation and landscape aesthetics)

CONAMA: National Environment Council of Brazil; COPAM: Environmental Policy Council of Minas Gerais; CERH: State Water Resources Council; FEPAM: Rio Grande do Sul Environmental Protection Foundation; CONEMA: Rio de Janeiro State Environmental Council; FATMA: Santa Catarina Foundation for the Environment; CEMA: Paraná State Environmental Council; SMA: São Paulo State Secretariat for the Environment; TF: Toxicity Factor; DWW: Dairy Wastewater

**Fig. 2:**
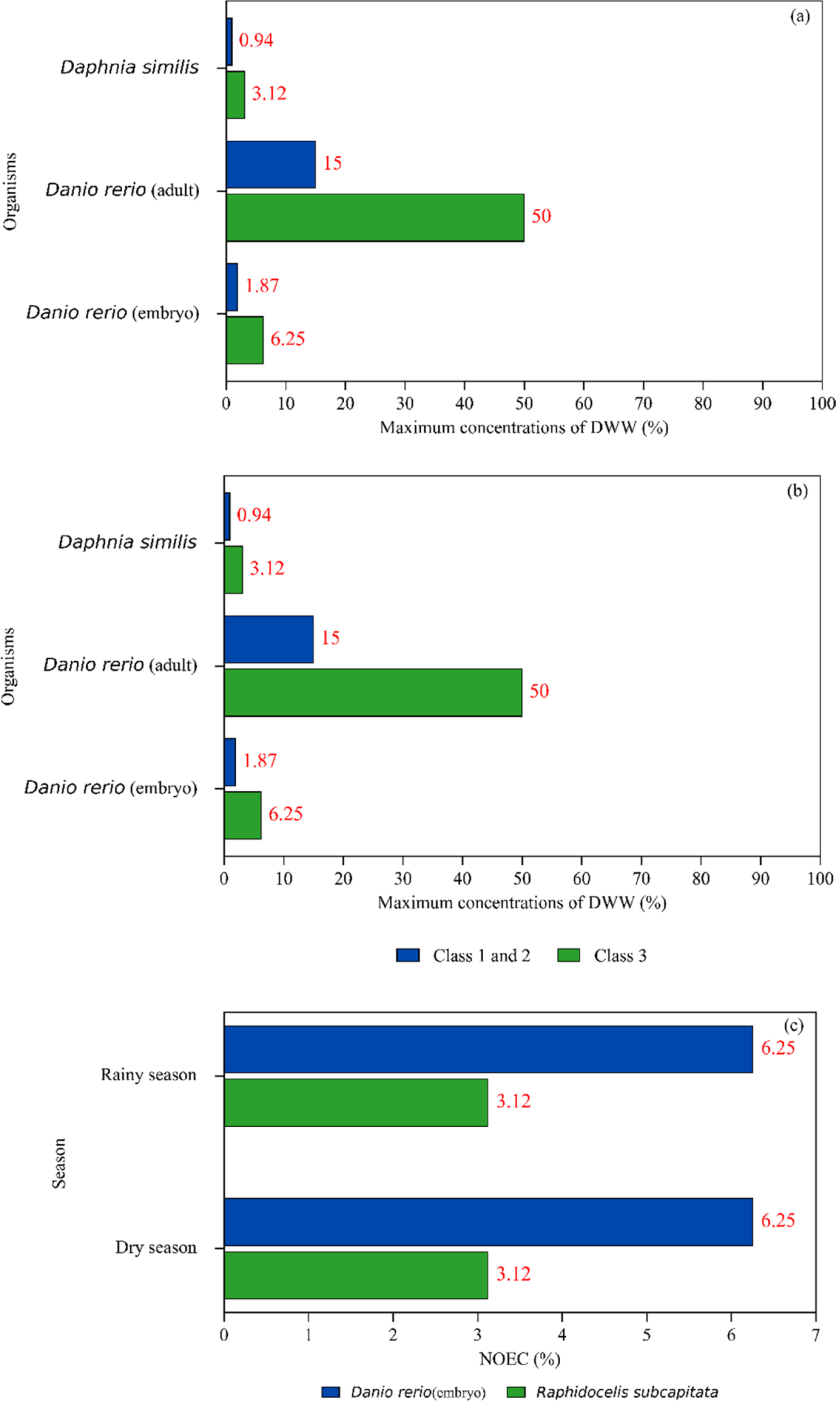
Maximum concentrations allowed for the discharge of the dairy wastewater under study considering limits established by Directives No. 430/2011 (Brasil, 2011) No. 357/2005 and for Class 1, 2 and 3 (Brasil, 2005): (**A**) acute ecotoxicity during the dry season, (**B**) acute ecotoxicity during the rainy season, and (**C**) chronic ecotoxicity during both rainy and dry season sampling campaigns

Figure 2 illustrates the maximum concentrations of DWW allowed in freshwater bodies as determined by the toxicity values from the case study for each test organism. These values were calculated based on the guidelines established in national regulations (Brasil, 2011). Disregarding the bioluminescent bacteria due to the incompatibility with DWW matrix, *Daphnia similis* was identified as the most restrictive test organism. The maximum concentration of DWW allowed in freshwater bodies (Classes 1 and 2, Brasil (2005) for *Daphnia similis* is 0.94%, meaning that the mixture must be diluted to 0.94% of the original effluent concentration (approximately a 106-fold dilution, v/v) to prevent acute effects. For Class 3 (Brasil, 2005) freshwater bodies, the minimum required dilution is 92.88%.

Moreover, as Classes 1 and 2 (Brasil, 2005) must not exhibit chronic toxicity (Brasil, 2011), the maximum permissible concentration of DWW to prevent chronic effects to *Raphidocelis subcapitata* is 3.12%, requiring a dilution factor of 96.86%. In the case of Class 3 (Brasil, 2005) freshwater bodies, no minimum dilution is required since there is no restriction for chronic toxicity (Brasil, 2011). However, under Directive No. 08/2022 (Minas Gerais, 2022), the maximum DWW concentration for Class 3 is aligned with those from Classes 1 and 2 (Brasil, 2005), indicating that state legislation is more protective of aquatic fauna than national directives.

The main concern regarding national regulation (Brasil, 2011), and state regulations in Minas Gerais (Minas Gerais, 2022), and Rio Grande do Sul (Rio Grande do Sul, 2017) is the lack of specific guidance regarding industrial typology concerning test organism selection and their respective toxicity factor threshold. This allows for the choice of ecotoxicological assays with resistant organisms, such as *Danio rerio* (adult) in the case of DWW analyzed in this case-study, potentially resulting in allowable DWW concentrations that are higher than protective levels. US and European directives show the same drawback as they do not specify toxicity tests that must be used for wastewater from each industry (EPA, 2000; OSPAR Commission, 2007).

Although more comprehensive, the CONEMA No. 86/2018 (Rio de Janeiro, 2018) directive and Decree FATMA No. 17/2002 (Santa Catarina, 2002) are still insufficient to control ecotoxicological risk from wastewater discharge. Both regulations propose specific test organisms and establish acute ecotoxicity limits for wastewater discharge. The Rio de Janeiro directive lists *Danio rerio*, *Pimephales promelas*, *Daphnia spp.*, *Aliivibrio fischeri* as test organisms with a maximum TF = 4, while the Santa Catarina legislation sets *Daphnia magna* (TF = 2) and *Aliivibrio fischeri* (TF = 4) for acute toxicity testing prior to discharge. However, these regulations allow for higher concentrations of wastewater discharge in freshwater bodies, particularly in Class 3 waterbodies (Brasil, 2005), than those assessed using the more sensitive organisms from this case study. This occurs because there is no requirement for chronic ecotoxicity assays, leaving only national directives to address this aspect. Moreover, according to this study, *Aliivibrio fischeri* is not a suitable test organism to assess the ecotoxicity of this wastewater matrix, as physicochemical characteristics of the wastewater (such as turbidity and color) influenced bioluminescence measurements. Consequently, while these regulations may seem restrictive, they fall short of providing comprehensive protection. This agrees with international regulations which do not list *Aliivibrio fischeri* as a standard assay for wastewater toxicity analysis (Norberg-King et al., 2018; EPA, 2000; Environment Canada, 2010; Goverment of Canada, 2012).

Finally, the Paraná and São Paulo directives are the most comprehensive among those listed in Table 4, establishing test organisms based on industrial wastewater typology for both acute and chronic ecotoxicity, as well as setting maximum TF required for wastewater discharge. The Paraná regulation CEMA No. 81/2010 (Paraná, 2010) specifies *D. magna* (TF = 2) and *Aliivibrio fischeri* (TF = 2) as test organisms for acute toxicity bioassays, while *Ceriodaphnia dubia* (TF = 2) and *Scenedesmus subspicatus* (TF = 2) are designated for chronic toxicity bioassays. Similarly, the São Paulo Directive SMA No. 003/2000 (São Paulo, 2000) defines *Daphnia similis* and *Aliivibrio fischeri* for acute toxicity assays, as well as *Ceriodaphnia dubia* and *Raphidocelis subcapitata* for chronic toxicity bioassays, while also stating that these should be determined on a case-by-case basis depending on the physicochemical characteristics of the wastewater. Although both regulations include *Aliivibrio fischeri* in their ecotoxicological assessments, they also require testing with other organisms, ensuring a more thorough evaluation of wastewater ecotoxicity. This approach allows for a more reliable ecotoxicological profile, addressing potential limitations associated with *Aliivibrio fischeri* ecotoxicity assessment alone.

## Conclusion

The dairy industry in Minas Gerais shows significant pollution potential to freshwater bodies due to the high number of facilities and substantial volumes of DWW discharged into watersheds. Environmental legislation is a critical tool for preventing and controlling pollution from anthropogenic activities, yet current Brazilian standards related to the ecotoxicity of industrial wastewater require updating to reflect the specific characteristics and ecological impacts of these discharges. The case study presented herein investigated acute and chronic effects of DWW from a facility that is typical of small dairy plants operating under simplified environmental licensing conditions in Minas Gerais and the surface water from the receptor water body. Although results cannot be generalized to all dairies operating under similar conditions, they provide a baseline that underscores the need for broader ecotoxicological assessments across multiple facilities. The challenges we encountered in gaining access to wastewater from multiple sites emphasize the importance of closer cooperation among academia, industry and regulatory agencies to enable robust sampling campaigns and to refine ecotoxicological assessment methods.

Physicochemical analysis revealed high values of COD and BOD_5_, indicating significant organic pollution potential. DWW also contained high concentrations of total nitrogen and phosphorus, exacerbating eutrophication of the receiving pond. Among the four different test organisms used to assess the ecotoxicity of DWW, *Daphnia similis* (acute toxicity) and *Raphidocelis subcapitata* (chronic toxicity) were the most sensitive test organisms. Notably, no toxicity to *Aliivibrio fischeri* was observed for the dry season sample, while samples collected in the rainy season showed high toxicity. This discrepancy underscores the need for more precise regulations that specify appropriate test organisms for different industrial wastewater matrices as four of the most comprehensive regulatory frameworks in Brazil list this bacterium as an appropriate test-organism to test acute toxicity of industrial wastewater.

Regarding current regulations on the ecotoxicology of industrial wastewater in Brazil, despite specific guidance by Directive CONEMA No. 86/2018 (Rio de Janeiro, 2018) and Decree FATMA No. 017/2002 (Santa Catarina, 2002), they fail to include maximum discharge calculations for chronic ecotoxicity evaluations. This oversight could result in inadequate protection for aquatic environments, emphasizing the need for a revision of these regulations to incorporate chronic ecotoxicity criteria. In contrast, Directive CEMA No. 81/2010 (Paraná, 2010) establishes protocols for both acute and chronic ecotoxicity assessments, alongside minimum toxicity factors for wastewater discharge into freshwater bodies. Such comprehensive measures may promote a more robust evaluation of DWW ecological impact. Directive SMA No. 003/2000 (São Paulo, 2000) offers a more comprehensive approach by requiring that each industrial wastewater should be evaluated on a case-by-case basis, further enhancing the assessment of environmental risks. Other current regulations, including CONAMA No. 430/2011 (Brasil, 2011) and COPAM/CERH No. 08/2022 (Minas Gerais, 2022), impose limits based on chronic and acute effects. Nonetheless, they lack the specificity necessary regarding the test organisms and allowable toxicity levels for each industrial wastewater matrix. This gap can lead to toxic conditions in surface waters and consequent ecological imbalances.

Findings obtained in this study underscore the urgent need to raise comprehensive data on acute and chronic toxicity to various test-organisms of DWW for the development and enhancement of regulatory standards that accommodate case-by-case analyses of wastewater impacts. By prioritizing the unique effects of DWW on sensitive organisms, regulatory frameworks can be improved to ensure effective environmental protection and the preservation of aquatic ecosystems. In summary, there is a critical need to update and refine environmental standards to meet the challenges posed by DWW, as well as other complex industrial wastewater, ultimately safeguarding ecological integrity.

## Supplementary Information

Below is the link to the electronic supplementary material.

Supplementary Material 1

## Data Availability

No datasets were generated or analysed during the current study.
