# Supplementary material for: Ecotoxicity assessment of dairy wastewater: environmental risks and regulatory needs
Source: Ecotoxicology. 2026 Apr 24;35(5):102. doi: 10.1007/s10646-026-03088-7 (PMC13109114; doi:10.1007/s10646-026-03088-7)
Supplement: Supplementary file 1 — Supplementary Material 1 [file 10646_2026_3088_MOESM1_ESM.docx]

**ECOTOXICITY ASSESSMENT OF DAIRY WASTEWATER: ENVIRONMENTAL RISKS AND REGULATORY NEEDS**

Magno Lange Albuquerque^1^, Fernando Rodrigues-Silva^1^, Alyson Rogério Ribeiro^2^, Carollina Maria Chávez^1^, Gilcinéa de Cássia Santana^3^, Alessandra da S. Martins^1^, Camila Costa de Amorim^1^, Maria Clara V. M. Starling^1*^

*Corresponding author: [mariaclara@desa.ufmg.br](mailto:mariaclara@desa.ufmg.br)

^1^Universidade Federal de Minas Gerais, Department of Sanitary and Environmental Engineering, Applied Ecotoxicology Group (ECOA), Av. Presidente Antônio Carlos 6627, Escola de Engenharia, Belo Horizonte 31270-901, MG, Brazil.

^2^Universidade Federal de Minas Gerais (UFMG), Department of Preventive Veterinary Medicine, Av. Presidente Antônio Carlos 6627, Escola de Veterinária, Belo Horizonte
31279-901, MG, Brazil.

^3^Universidade Federal de Minas Gerais, Department of Veterinary Clinic and Surgery, Av. Presidente Antônio Carlos 6627, Escola de Veterinária, Belo Horizonte
31279-901, MG, Brazil.

# MATERIALS AND METHODS

## CASE STUDY: DAIRY INDUSTRY

The production process follows a traditional method of fresh cheese manufacturing and is similar to the one described by Santos et al. (2017). The first stage is raw milk filtration followed by pasteurization and standardization (milk is heated to 62 ºC for 30 minutes). Pasteurized milk is then cooled down to 30 ºC. Salt and coagulant agent are added to the pasteurized milk, and the mixture is set to rest for 50 min. Finally, the cheese is salted, molded, and taken to a freezer to be packed the next day (Figure S1A). For yogurt production, the mixture is homogenized after raw milk pasteurization and set to approximability 40 ºC, the appropriate temperature for the growth of starter cultures. Afterwards, starter cultures (*Lactobacillus bulgaricus* and *Streptococcus thermophilus*) are added to the pasteurized milk. When the pH reaches 4.5, the mixture is set to 7 ºC, it is flavored and packed (Giner Santoja et al., 2019a) (Figure S1B).

Figure S1 - Flowchart showing production stages and main sources of wastewater in the production of (A) cheese and (B) yogurt from raw milk.


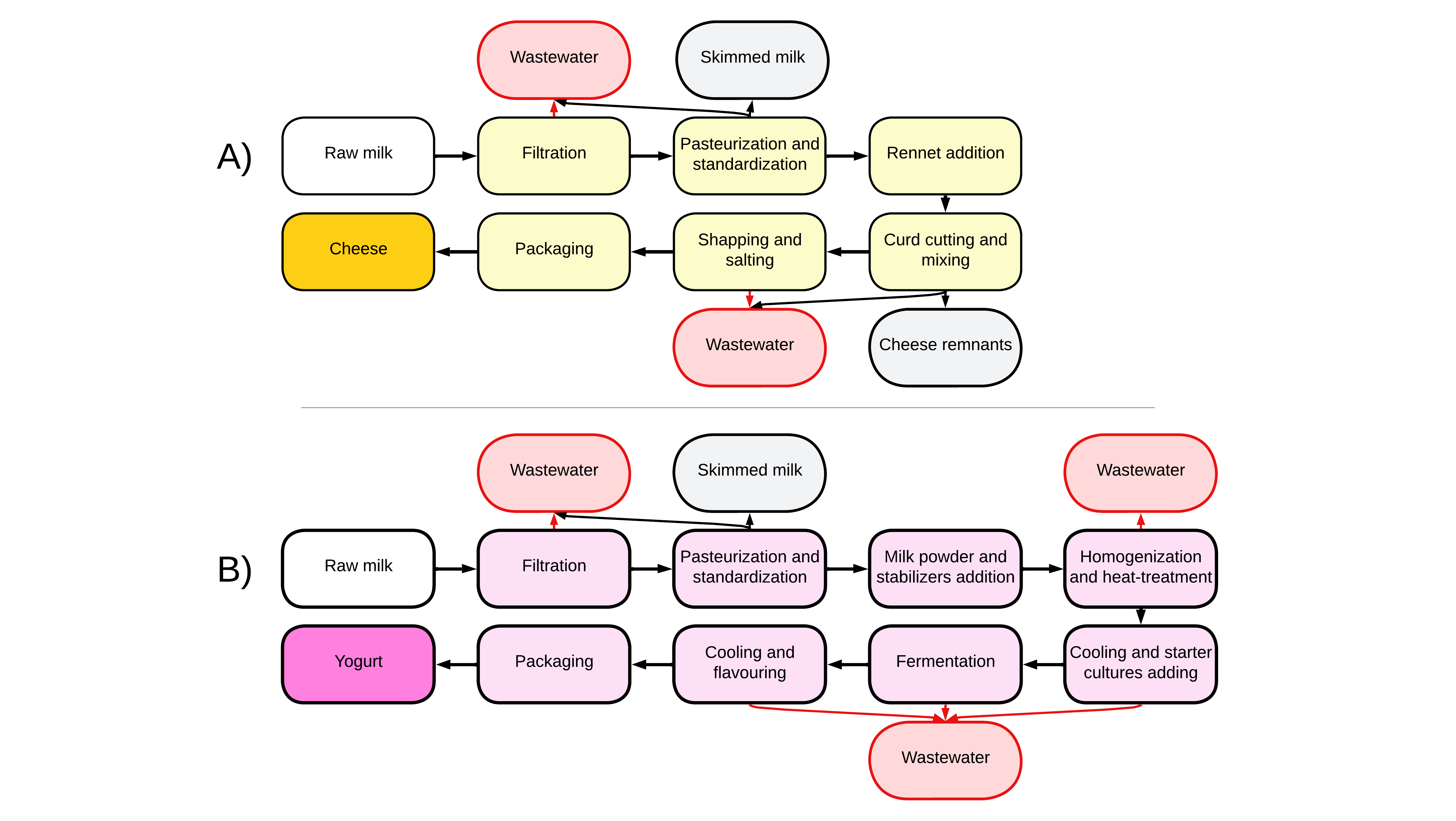


Source: Adapted from H. C. M. Santos et al. (2017) and Giner Santoja et al. (2019).

For this industry, DWW generation (Eν) was estimated according to Equation S1 (Von Sperling, 2014). Processed milk average density (ρ) was considered as 1.02772 kg L^-1^ (Rahmawati & Juwitaningtyas, 2024), wastewater production (ν) was estimated as 2.5 L kg­^-1^ of processed milk, and the milk processing rate (Р) is 1200 L per day^-1^. Hence, the dairy industry generates approximately 3,083.16 L of DWW per day^-1^.

|  | $E\nu= \rho\times\nu\timesР$ | (1) |
| --- | --- | --- |

## CRITICAL ANALYSIS OF CURRENT TOXICITY STANDARDS

For water bodies classified as Classes 1 and 2 (Brasil, 2005), the maximum wastewater concentration (Equation S3) allowed must be less than or equal to the NOEC derived from a chronic toxicity test. For acute toxicity assessment, Equations S4 and S5 determine the maximum wastewater concentration allowed. However, for Class 3 freshwater bodies (Brasil, 2005), Equations S6 and S7 are used to assess the maximum allowable wastewater concentration. Additionally, Directive FATMA No. 17/2002 (Santa Catarina, 2002) determines the maximum wastewater concentration in freshwater bodies based on TF values from acute toxicity responses (Equation S8).

|  | $WC \left( \% \right)=\frac{Q_{Wastewater}\times100}{Q_{Wastewater}+Q_{7,10}}$ |  | (S3) |
| --- | --- | --- | --- |
|  | $WC(\%)\leq\frac{30}{TF}$ |  | (S4) |
|  | $WC(\%)\leq\frac{{LC}_{50}}{10}$ |  | (S5) |
|  | $WC(\%)\leq\frac{100}{TF}$ |  | (S6) |
|  | $WC(\%)\leq\frac{{LC}_{50}}{3}$ |  | (S7) |
|  | $WC(\%)\leq\frac{{100}/{TF}}{2}$ |  | (S8) |

Where WC is wastewater concentration in the recipient water body (%), Q_Wastewater_ is the maximum daily wastewater discharge (m^3^ per day^-1^), and Q_7,10_ is the annual average of the daily flow that occurs on average only once every ten years (m^3^ per day^-1^).

# Control charts

Figure S2 – Control chart for *Raphidocelis subcapitata* - sensitivity assays using Sodium Chloride (NaCl).


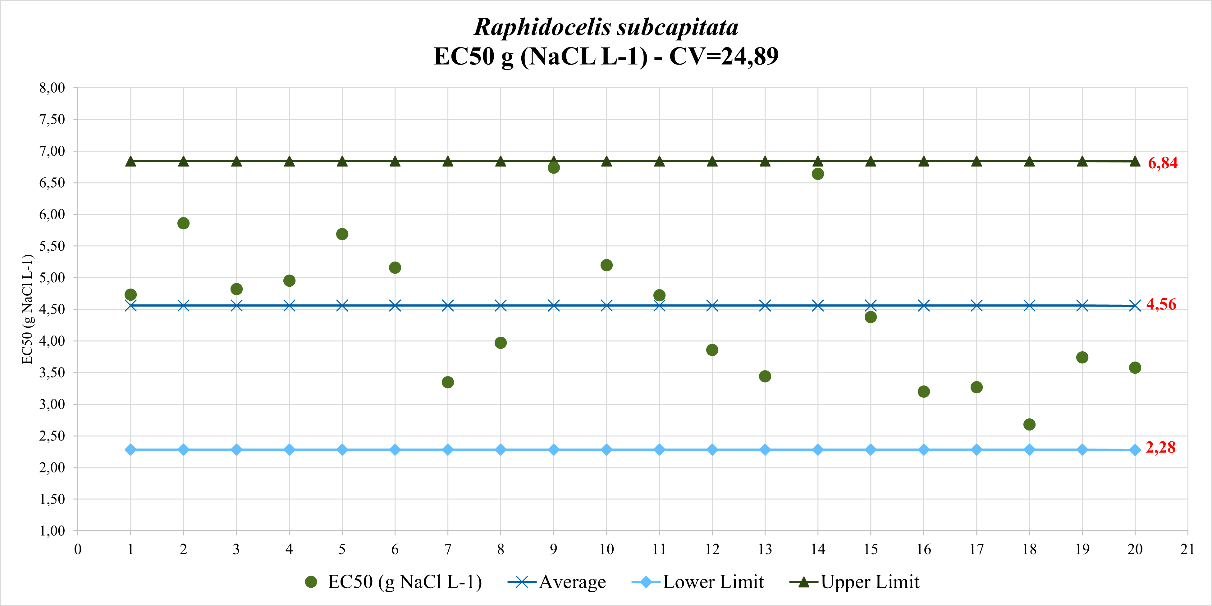


Figure S3 – Control chart for *Daphnia similis* - sensitivity assays using Sodium Chloride (NaCl)


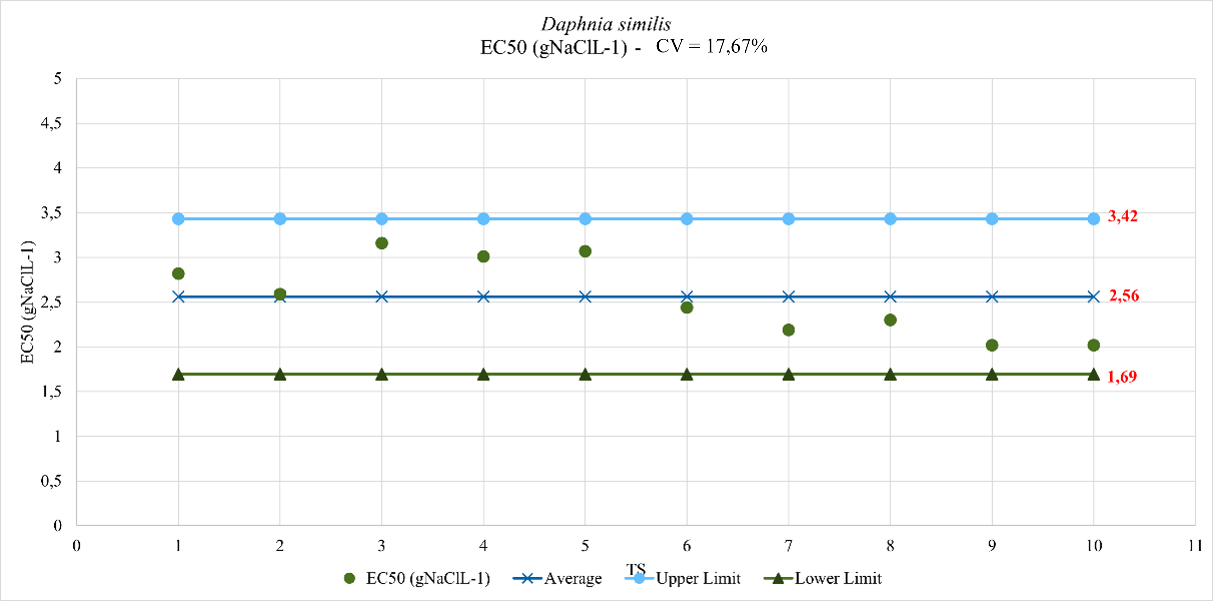


Figure S4 – Control chart for *Danio rerio* (larval) - sensitivity assays using Sodium Chloride (NaCl).


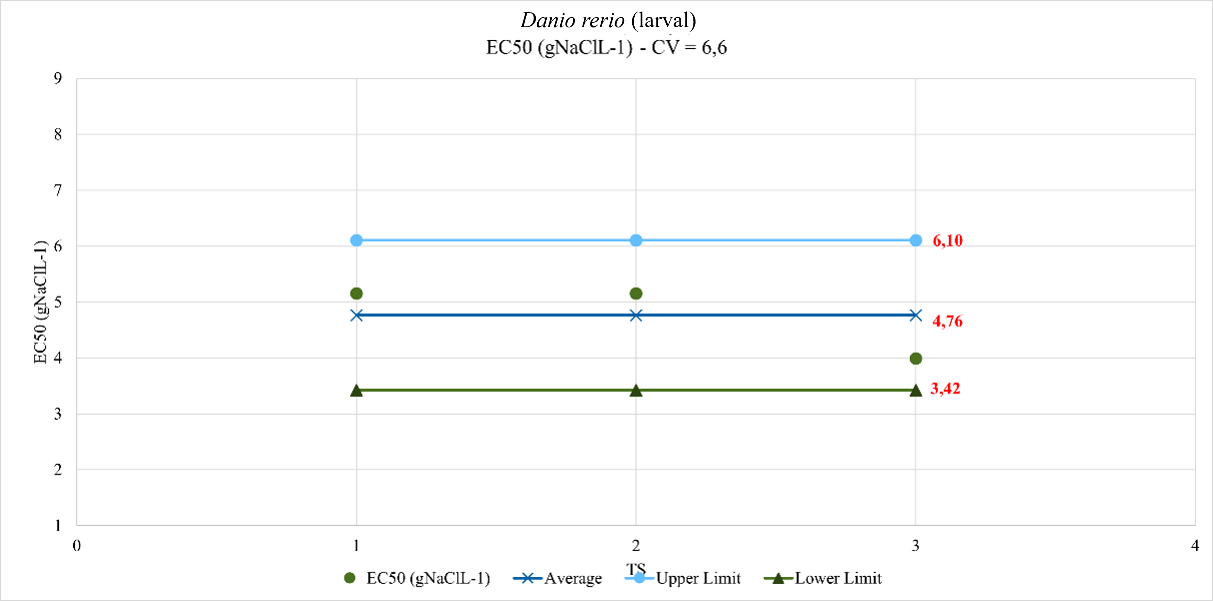


Figure S5 – Control chart for *Danio rerio* (adult) - sensitivity assays using Sodium Chloride (NaCl).


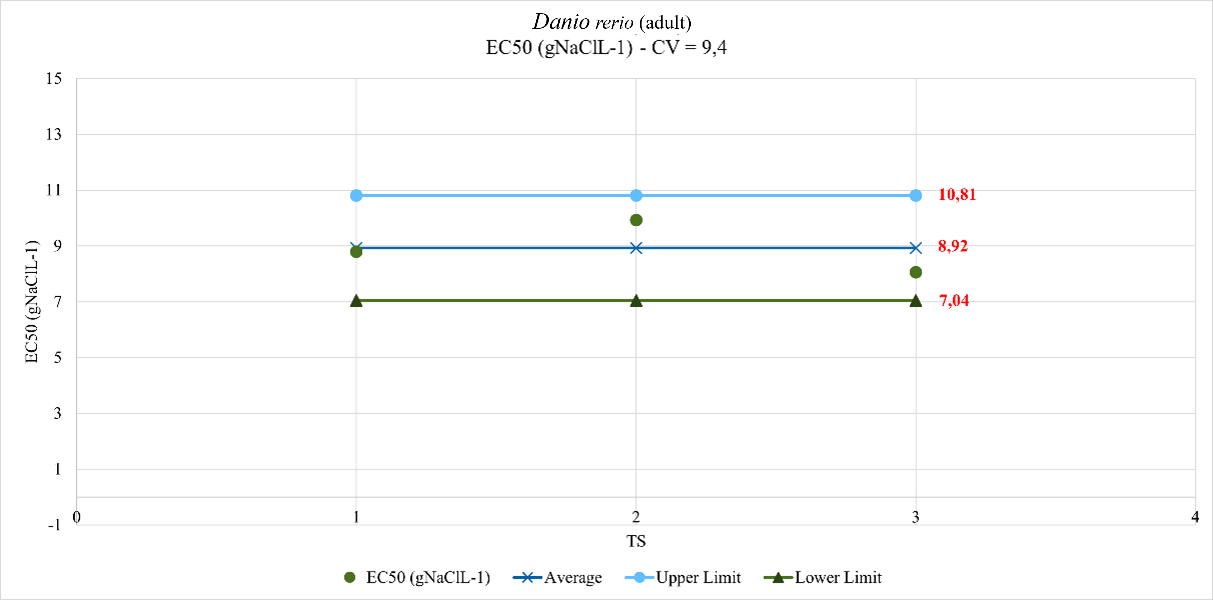


Figure S6 – Control chart for *Aliivibrio fischeri* - sensitivity assays using K_2_Cr_2_O_7_.


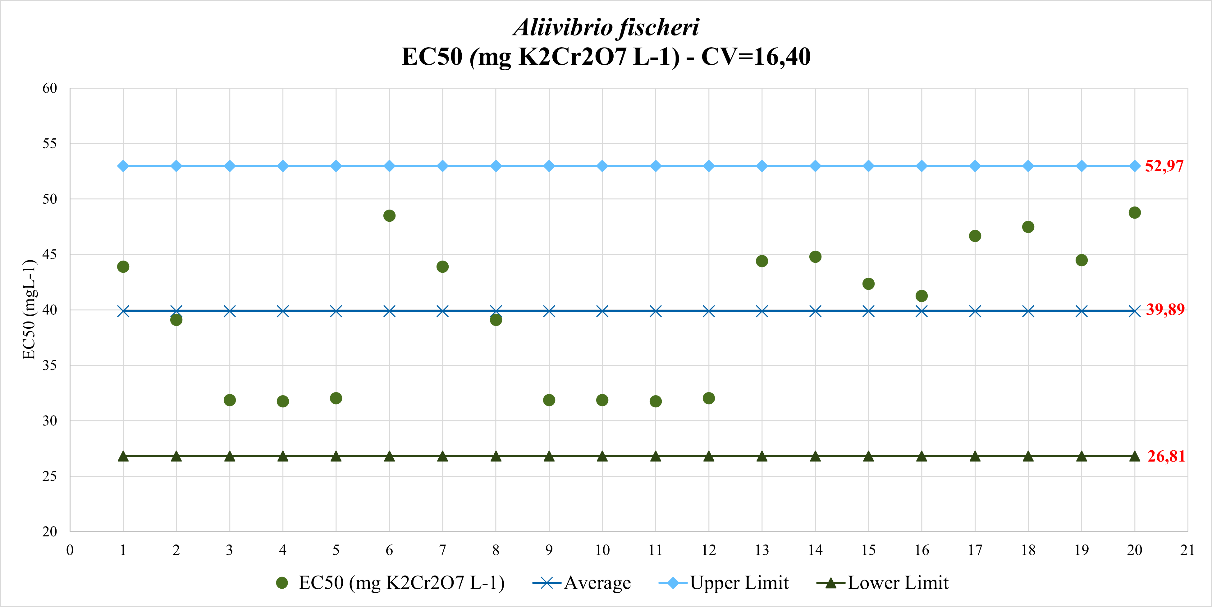


**REFERENCES**

ABNT. (2021a). Associação Brasileira de Normas Técnica - Ecotoxicologia aquática — Efeito inibitório sobre a bioluminescência de Vibrio fscheri Parte 3: Método utilizando bactérias lioflizadas. Em *15411-3*.

ABNT. (2022a). Associação Brasileira de Normas Técnicas - ABNT NBR 12713/2022 - Ecotoxicologia aquática - Toxicidade aguda - Método de ensaio com Daphnia spp (Crustacea,Cladocera). Em *12713/2022*.

ABNT. (2022b). Associação Brasileira de Normas Técnicas - ABNT NBR 15088/2022 - Ecotoxicologia aquática - Toxicidade aguda - Método de ensaio com peixes (Cyprinidae). Em *15088/2022*.

ABNT. (2022c). Associação Brasileira de Normas Técnicas - ABNT NBR 15499/2022 - Ecotoxicologia aquática — Toxicidade crônica de curta duração — Método de ensaio com peixes. Em *ABNT NBR 15499* (15499/2022).

ABNT. (2023). Associação Brasileira de Normas Técnicas - ABNT NBR 12648/2023 - Ecotoxicologia aquática - Toxicidade crônica - Método de ensaio com algas (Chlorophyceae). Em *12648/2023*.
